# Effects of synergistic/antagonistic interactions between spermidine (3+) and putrescine (2+) on gene expression through higher-order DNA structural modification

**DOI:** 10.1007/s00249-026-01831-w

**Published:** 2026-03-02

**Authors:** Haruto Ogawa, Takashi Nishio, Yuko Yoshikawa, Takahiro Kenmotsu, Kenichi Yoshikawa

**Affiliations:** 1 Faculty of Life and Medical Sciences, Doshisha University, Kyoto, 610- 0394 Japan; 2 Molecular Biosystems Research Institute, National Institute of Advanced Industrial Science and Technology (AIST), Ibaraki, 305-8566 Japan

**Keywords:** Higher-order structure of DNA, Single-molecular observation, Cell-free gene expression, Polyamine, Synergistic and antagonistic effects, Structure-activity relationship

## Abstract

Polyamines are essential cationic biomolecules that interact with negatively charged biomolecules, such as DNA, RNA, and proteins, thereby modulating their structure and function. Although spermidine(3+) and spermine(4+) often act synergistically, studies have indicated that putrescine(2+) can act antagonistically toward spermidine and other polyamines. To elucidate the associated synergistic/competitive effects, we investigated how putrescine and spermidine, when present together, cause higher-order structural changes in DNA and gene expression. Cell-free luciferase assays revealed that putrescine and spermidine exerted either synergistic or antagonistic effects on gene expression depending on their concentration. Millimolar concentrations of putrescine increased gene expression when combined with spermidine, but higher concentrations (e.g., 10 mM) inhibited expression. Atomic force microscopy revealed that structural changes in DNA were linked to changes in gene expression activity. These characteristic effects on DNA conformation and gene expression caused by the coexistence of putrescine and spermidine are examined in terms of physicochemical correlations among charged species, including the higher-order structure of negatively charged DNA, polycationic polyamines, and small counterions. At relatively low concentrations of polyamines, the effective negative charge of DNA decreases because of the cooperative effect between putrescine and spermidine. In contrast, at higher concentrations, the antagonistic effect becomes apparent as competitive interaction arises through counterion translational entropy. Notably, such bimodal effects could constitute a relatively general mechanism in the solution environment of living matter.

**Supplementary Information:**

The online version contains supplementary material available at https://doi.org/10.1007/s00249-026-01831-w.

## Introduction

Biological polyamines are polyvalent cations present in all living organisms. Although a wide variety of natural polyamines exist, diamine putrescine (PUT), triamine spermidine (SPD), and tetramine spermine (SPM) are among the most common ones and participate in important biological activities of viruses, bacteria (eubacteria and archaebacteria), and eukaryotes (Thomas and Thomas 2001; Childs et al. 2003; Wallace et al. 2003; Brooks 2013; Mandal et al. 2013; Weiger and Hermann 2014; Miller-Fleming et al. 2015; Pegg 2016; Lenis et al. 2017; Sagar et al. 2021; Zahedi et al. 2022; Xuan et al. 2023). These polyamines are essential for cell growth and proliferation. A common feature of polyamines is their cationic nature, which enables them to interact with negatively charged macromolecules, such as DNA, RNA, and proteins, thereby regulating their structure and function (Yamasaki et al. 2001; Vijayanathan et al. 2001; Saminathan 2002; Korolev 2003; Terui et al. 2005; Cherstvy and Petrov 2014; Igarashi and Kashiwagi 2015, 2019; Perepelytsya et al. 2019). PUT, SPD, and SPM are synthesized in vivo via a common polyamine metabolic pathway and exhibit common cationic properties with different numbers of positive charges. However, previous studies have revealed antagonistic effects of PUT against SPD and SPM (Rosano and Hurwitz 1977; Davis et al. 1985; Mills 1998; Seiler et al. 1998). SPD and SPM increase N-methyl-*D*-aspartate (NMDA) receptor activity, whereas PUT reduces receptor activity (Rock and MacDonald 1992; Igarashi and Williams 1995). Similar enhancements by SPD and SPM and inhibition by PUT have been observed for the binding of several proteins to DNA (Panagiotidis et al. 1995).

Polyamine-induced DNA compaction/condensation has been studied for more than half a century (Gosule and Schellman 1976; Chattoraj et al. 1978; Wilson and Bloomfield 1979; Tongu et al. 2016; Muramatsu et al. 2016; Kanemura et al. 2018; Zinchenko et al. 2018; Nishio et al. 2018, 2019, 2020, 2023; Kashiwagi et al. 2019; Tanaka et al. 2020; Kitagawa et al. 2021). We previously investigated the correlation between higher-order structural changes in genome-sized DNA induced by biological polyamines, particularly SPD, and gene expression in cell-free systems using single-molecule observations with fluorescence microscopy and atomic force microscopy (AFM) (Kanemura et al. 2018; Nishio et al. 2019, 2023; Kitagawa et al. 2021). In these studies, we reported that polyamines exert biphasic effects on gene expression; they increase gene expression when present at low concentrations but inhibit gene expression at high concentrations. Notably, a flower-like structure characterized by a unique shrunken conformation and a parallel alignment of DNA strands was observed at intermediate concentrations, which promoted gene expression. In contrast, tightly compacted DNA conformations were observed in the presence of higher concentrations of polyamines, and gene expression was completely inhibited (Kanemura et al. 2018; Nishio et al. 2019, 2023; Kitagawa et al. 2021). In addition, we reported that higher-valence polyamines induced folding transitions and inhibited gene expression at lower concentrations (Basu et al. 1990; Takahashi et al. 1997; Kanemura et al. 2018). In contrast, the coexistence of divalent metal cations, Mg(2+) and Ca(2+), and SPD has been shown to inhibit the ability of genome-sized DNA to induce folding transition (Tongu et al. 2016), at concentrations lower than that for the trivalent SPD to cause DNA compaction in the absence of divalent cations. It is noted that suppression and promotion of DNA charge inversion caused by the mixing effect of counter cations with different valencies have been observed through the measurements of electrophoretic mobility (Qiu et al. 2015; Wang et al. 2018). Despite several reports of antagonism of multiple cations of different valencies with respect to the property of DNA, its role in gene activity in the presence of polyamines with different valences has not yet been fully elucidated. Therefore, in this study, we examined the effect of PUT on SPD-induced conformational changes in genome-sized DNA and the resulting impact on gene expression in a cell-free system. Interestingly, we observed competitive (antagonistic) and cooperative (synergistic) effects between PUT and SPD on the conformation and activity of DNA, depending on their concentrations. Studying the bimodal effects of coexisting polyamines with different valences can elucidate the underlying mechanisms of complex biological reaction networks (Araujo and Liotta 2023; Dastan et al. 2024; Singh 2024; Roy et al. 2025) in living cells.

## Results

### Gene expression activity under the coexistence of SPD and PUT

**Fig. 1:**
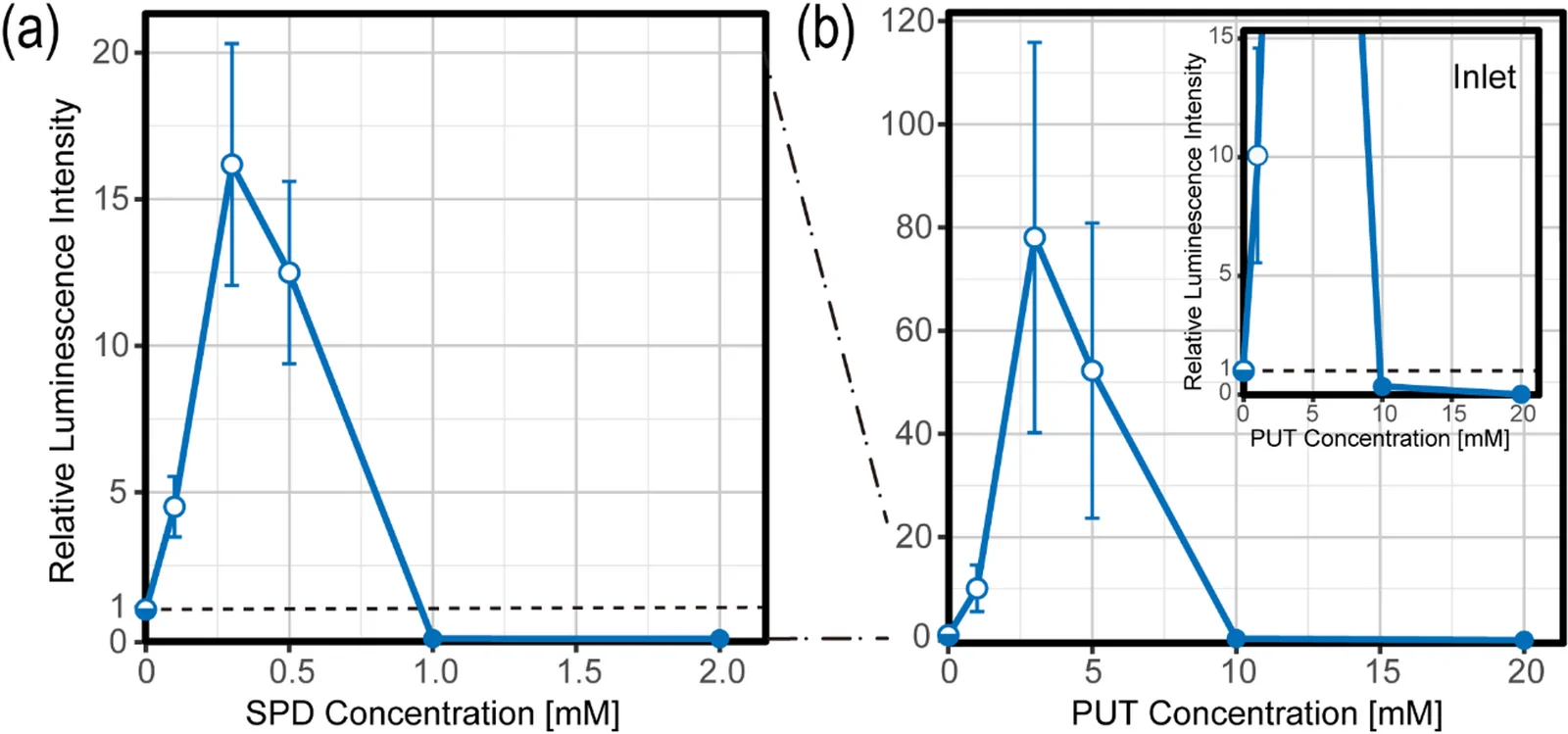
Efficiency of gene expression depending on the concentrations of (**a**) spermidine (SPD) and (**b**) putrescine (PUT). The vertical axis shows the relative emission intensity of the luciferin–luciferase reaction, normalized to that of the control (1.00), where SPD and PUT were absent. The data are shown as the means ± standard deviations (SDs) of at least three independent experiments. The DNA concentration was fixed at 0.6 µM in the nucleotide units

We explored the effects of SPD and PUT on cell-free gene expression via a luciferase assay. The relative luminescence intensities of the luciferin–luciferase reaction as a marker of gene expression in the presence of (a) SPD or (b) PUT are shown in Fig. 1. The intensities were normalized on the basis of those of the control experiments in the absence of SPD and PUT. The two polyamines promoted and inhibited gene expression in a biphasic manner, depending on their concentrations. Figure 1(a) shows that gene expression activity was maximally promoted by SPD at concentrations ranging from 0.3 to 0.5 mM and completely inhibited beyond 1.0 mM, which is consistent with the results of previous studies (Kanemura et al. 2018; Nishio et al. 2019, 2020). However, gene expression was maximally promoted by PUT at concentrations ranging from 3 to 5 mM and completely inhibited at concentrations above 10 mM, which is consistent with the results of a previous study (Tanaka et al. 2020). Interestingly, PUT promoted expression activity approximately 2–5 times more notably than did SPD.

**Fig. 2:**
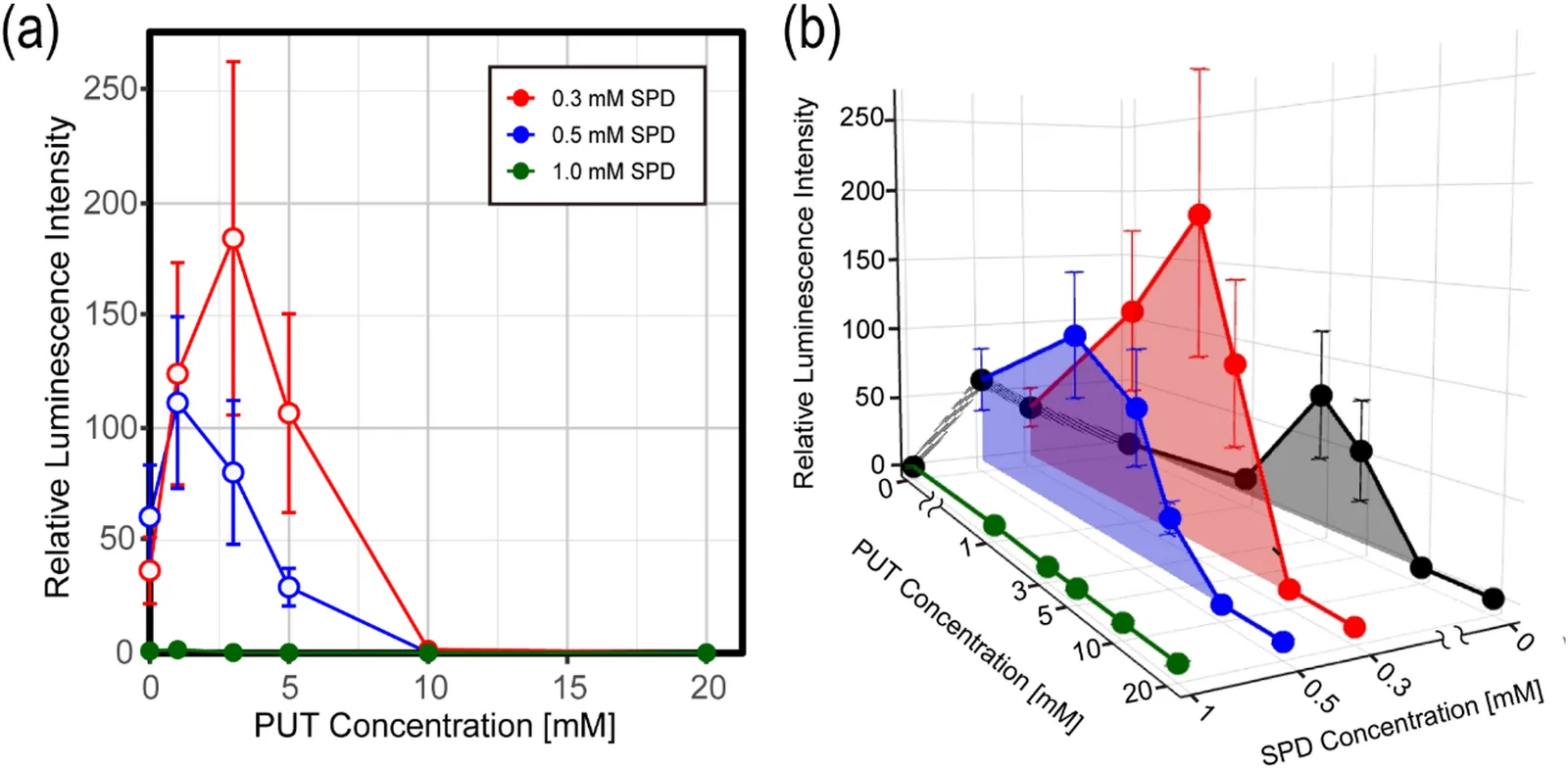
Effects of the coexistence of PUT and SPD on gene expression. (**a**) Efficiency of gene expression in the presence of 0.3, 0.5, and 1.0 mM SPD at various concentrations of PUT and (**b**) its quasi-3D representation, with the addition of the data at 0 mM SPD shown in Fig. 1(b). The vertical axis is the relative emission intensity of the luciferin–luciferase reaction, normalized to that of the control (1.00), where SPD and PUT were absent. The data are shown as the means ± SDs of at least three independent experiments. The DNA concentration was fixed at 0.6 µM in the nucleotide units

Next, we investigated the effect of PUT on gene expression activity in the presence of SPD (0.3, 0.5, and 1.0 mM) under the characteristic concentrations that promote (0.3, 0.5 mM) and inhibit (1.0 mM) gene expression activity, as shown in Fig. 1. As shown in Fig. 2, PUT promoted gene expression even in the presence of 0.3 and 0.5 mM SPD, but above 10 mM, PUT completely inhibited gene expression, as well as in the absence of SPD. However, gene expression activity was consistently inhibited even when PUT was applied at the SPD inhibition concentration (1.0 mM). The decrease in the maximum relative luminescence intensity and shift in the maximum concentration promoting gene expression toward a lower concentration revealed that gene expression activity under coexisting conditions was inhibited by increasing the SPD concentration.

### Higher-order structure of DNA under the coexistence of SPD and PUT

**Fig. 3:**
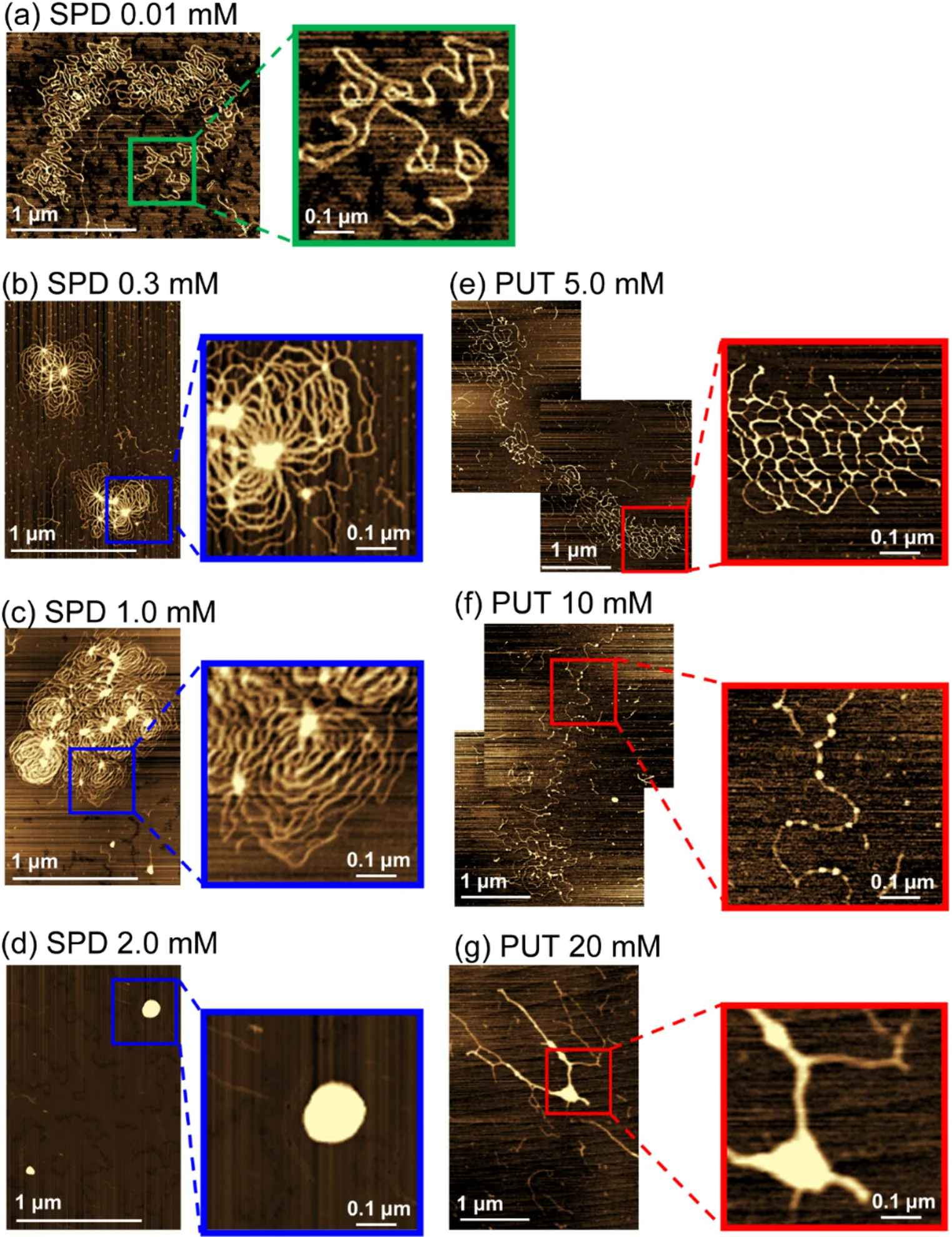
AFM images of T4 GT7 DNA at various concentrations of SPD and PUT: (**a**) 0.01 mM SPD; (**b**) 0.3 mM SPD; (**c**) 1.0 mM SPD; (**d**) 2.0 mM SPD; (**e**) 5.0 mM PUT; (**f**) 10 mM PUT; and (**g**) 20 mM PUT. Expanded images are shown on the right side of the micrometer-scale AFM images. The DNA concentration was fixed at 0.6 µM in the nucleotide units

We observed T4 GT7 DNA (166 kbp) via AFM at various SPD and PUT concentrations to determine the effects on the higher-order structure of DNA. Representative AFM images of DNA in the presence of different concentrations of SPD ((a) 0.01 mM, (b) 0.3 mM, (c) 1.0 mM, and (d) 2.0 mM) and PUT ((e) 5.0 mM, (f) 10 mM, and (g) 20 mM) are shown in Fig. 3 (refer also to Fig. S1). As shown in Fig. 3(a), the T4 GT7 DNA was in the elongated coil form at 0.01 mM SPD. Here, we adapted the experimental conditions to use a DNA solution with a very low concentration of SPD (0.01 mM), as shown in Fig. 3(a), to allow DNA to adsorb onto the mica surface. With increasing SPD concentration, the DNA formed a flower-like structure, which caused the loose shrinking of the DNA conformation with parallel-aligned segments, at 0.3 and 1.0 mM SPD, as shown in Fig. 3(b) and (c), respectively, and was tightly compacted at 2.0 mM SPD (Fig. 3(d). These results are consistent with those of previous studies (Kanemura et al. 2018; Nishio et al. 2018, 2019). A comparison of the flower-like structures at 0.3 and 1.0 mM SPD revealed that the number of highly condensed small DNA cores increased with increasing SPD concentration and that the spacing between DNA segment sequences tended to narrow. The contrast represented in the AFM images corresponds to the surface height. Therefore, regions with higher contrast indicated highly condensed DNA regions.

Interestingly, the higher-order structural changes induced by PUT are highly different from those induced by SPD. With increasing PUT concentration, the DNA formed mesh-like (5.0 mM PUT), partially thick (10 mM PUT), and kinked branching (20 mM PUT) conformations (Fig. 3(e)–(g). Interestingly, exposure to PUT caused a mesh-like cross-linking conformation, without the formation of a flower-like structure or a highly condensed structure, which are observed in the presence of polyamines with higher cationic numbers, such as SPD, SPM, and homocaldopentamine(5+) (Tongu et al. 2016; Muramatsu et al. 2016; Kanemura et al. 2018; Nishio et al. 2018, 2019, 2020, 2023; Kitagawa et al. 2021).

**Fig. 4:**
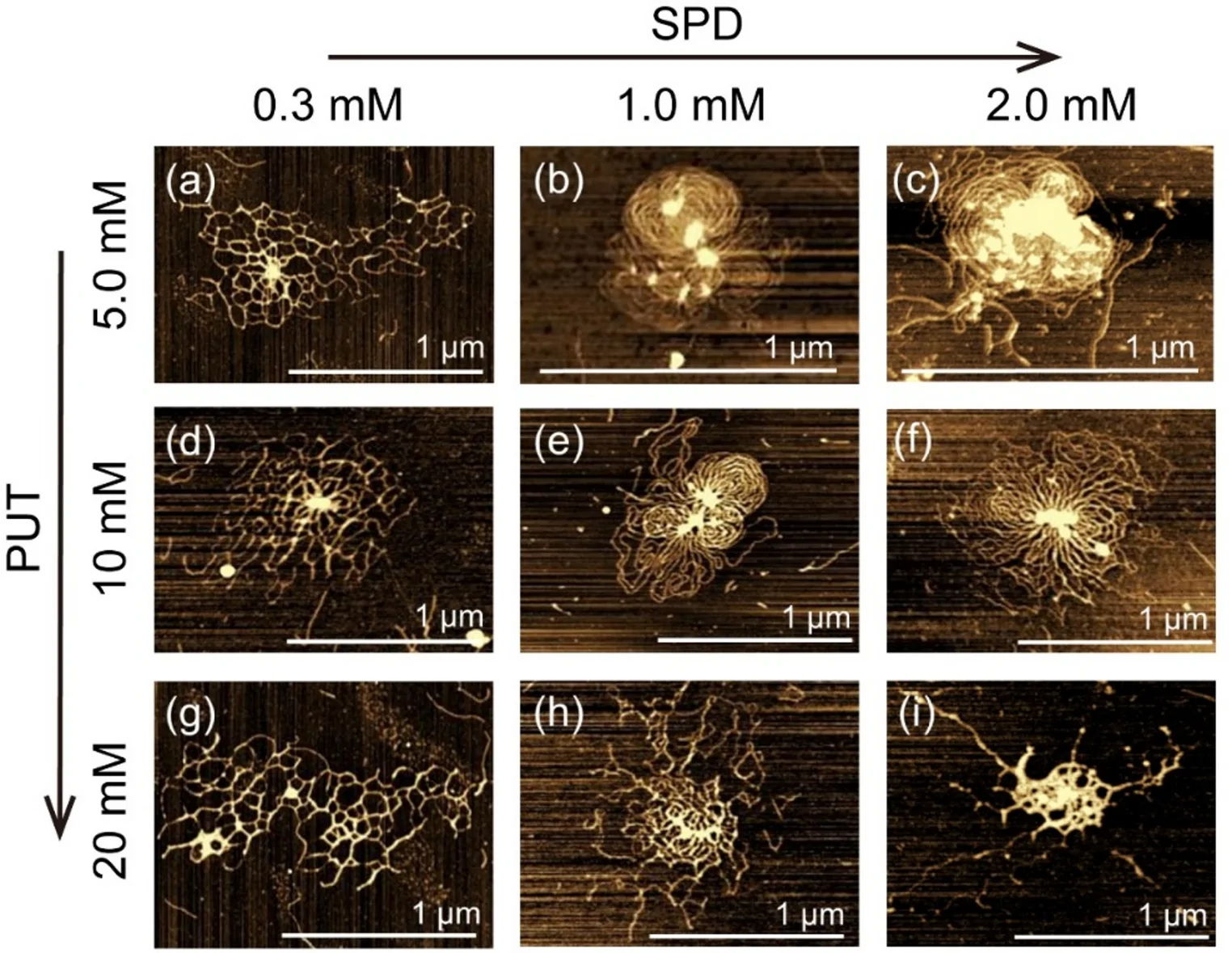
AFM images of T4 GT7 DNA under the coexistence of SPD and PUT. The SPD concentrations were fixed at 0.3, 1.0, and 2.0 mM in each column, and the PUT concentrations were fixed at 5.0, 10, and 20 mM in each row. The DNA concentration was fixed at 0.6 µM in the nucleotide units

AFM images representing the effect of PUT on the higher-order structure of DNA in the presence of 0.3, 1.0, and 2.0 mM SPD are shown in Fig. 4. As shown in Fig. 4(a)(d)(g), under a fixed 0.3 mM SPD concentration with PUT (5.0, 10, and 20 mM), cross-liking mesh-like structures appeared, which are similar to the DNA conformations with 5.0 mM PUT in the absence of SPD (Fig. 3(e)), in contrast to the generation of a flower-like structure with 0.3 mM SPD in the absence of PUT (Fig. 3(b)). The mesh-like structure remains in a similar state with increasing PUT concentration, revealing that the presence of 0.3 mM SPD induces the emergence of a mesh-like conformation, which is different from the observations without SPD (Fig. 3(f)(g)). With respect to the observations with the addition of PUT in the presence of 1.0 mM SPD, DNA maintained the flower-like structure for 0 mM PUT (Fig. 3(c)), 5.0 mM PUT (Fig. 4(b)), and 10 mM PUT (Fig. 4(e)), but the parallel alignment of DNA strands was no longer maintained at 20 mM PUT (Fig. 4(h)). Interestingly, in the presence of 2.0 mM SPD, the highly compact state (without PUT; Fig. 3(d)) unfolded and exhibited swelling under the addition of PUT (5.0 mM in Fig. 4(c)), and the parallel alignment of DNA strands was no longer maintained (10 mM in Fig. 4(f) and 20 mM in Fig. 4(i)).

### Quantitative evaluation of the higher-order structural change in DNA caused by SPD or PUT

The experimental results revealed that polyamines with different valences induced distinct higher-order structural changes in DNA. SPD induced a flower-like structure with a parallel alignment of DNA segments and caused tight DNA compaction at higher concentrations. In contrast, PUT caused the formation of a mesh-like structure with a more swollen conformation. At higher PUT concentrations, bundling of DNA segments was observed. Notably, PUT exhibited the ability to unfold the tightly compacted state of DNA in the presence of a high concentration (2.0 mM) of SPD, but compaction similar to that induced by SPD was not observed. Such distinct effects on the higher-order structure of DNA can be of concern for gene expression in cell-free systems.

**Fig. 5:**
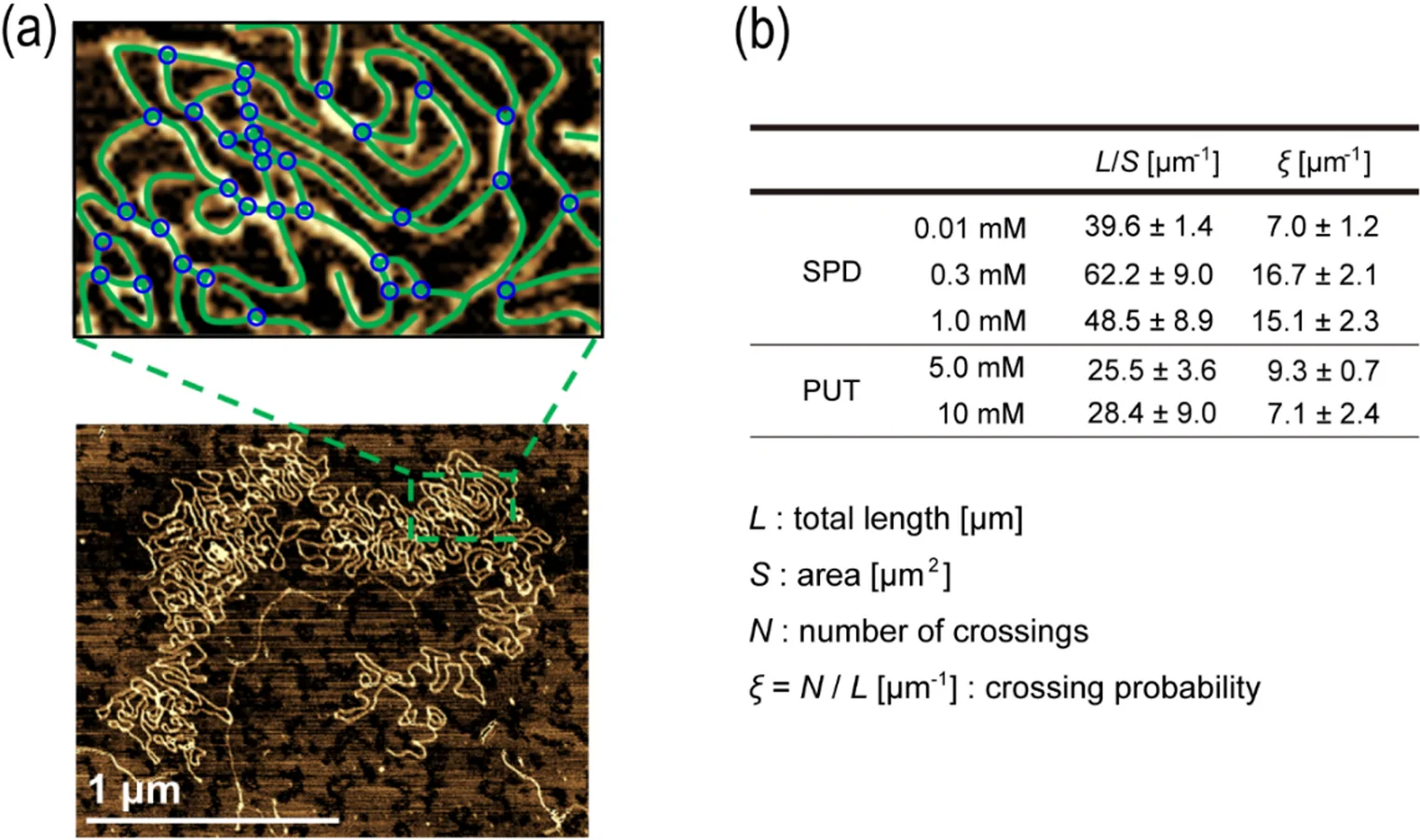
Segmental density, *L*/*S*, and crossing probability, *ξ*, evaluated from the AFM images. (**a**) Example of the procedure for evaluating the *L*/*S* and *ξ* from the AFM images of T4 GT7 DNA shown in Fig. 3(a) and S1 (**a**). The average values with SD obtained from the analyses of three individual DNA molecules, which have a major structural feature under each condition, are summarized in the table shown in (**b**) on the right

To quantitatively evaluate the differences in higher-order DNA structures induced by SPD and PUT, we evaluated the segmental density (*L*/*S*, µm^− 1^) and the crossing probability *ξ* (= *N*/*L*, µm^− 1^) from the AFM images. These values were obtained by measuring the total DNA length (*L*, µm) within a fixed area (*S*, µm^2^) and counting the number of crossings (*N*) within the length *L* (Fig. 5, Table S1). As a result, compared with the coiled state observed at 0.01 mM SPD, the flower-like structures formed at 0.3 and 1.0 mM SPD exhibited more than twofold greater *ξ* values (Fig. 5(b), accompanied by an increase in *L/S*. These results indicate that the flower-like structure caused by the addition of SPD has a more shrunken conformation than the coil state does with increasing probability of crossing. Such a characteristic higher-order structure of DNA is expected to be formed through the decrease in the negative charges of phosphate groups caused by SPD, which promotes cross-linking between DNA segments, while the residual negative charges still generate electrostatic repulsion (Kanemura et al. 2018). This structural arrangement, accompanied by a decrease in the negative charge along the DNA chain, likely facilitates interactions with RNA polymerase, which also has a net negative charge, thereby increasing gene expression. The observed changes in the higher-order structure of DNA are consistent with the results shown in Fig. 1(a), where gene expression was significantly enhanced in the presence of 0.3–0.5 mM SPD.

**Fig. 6:**
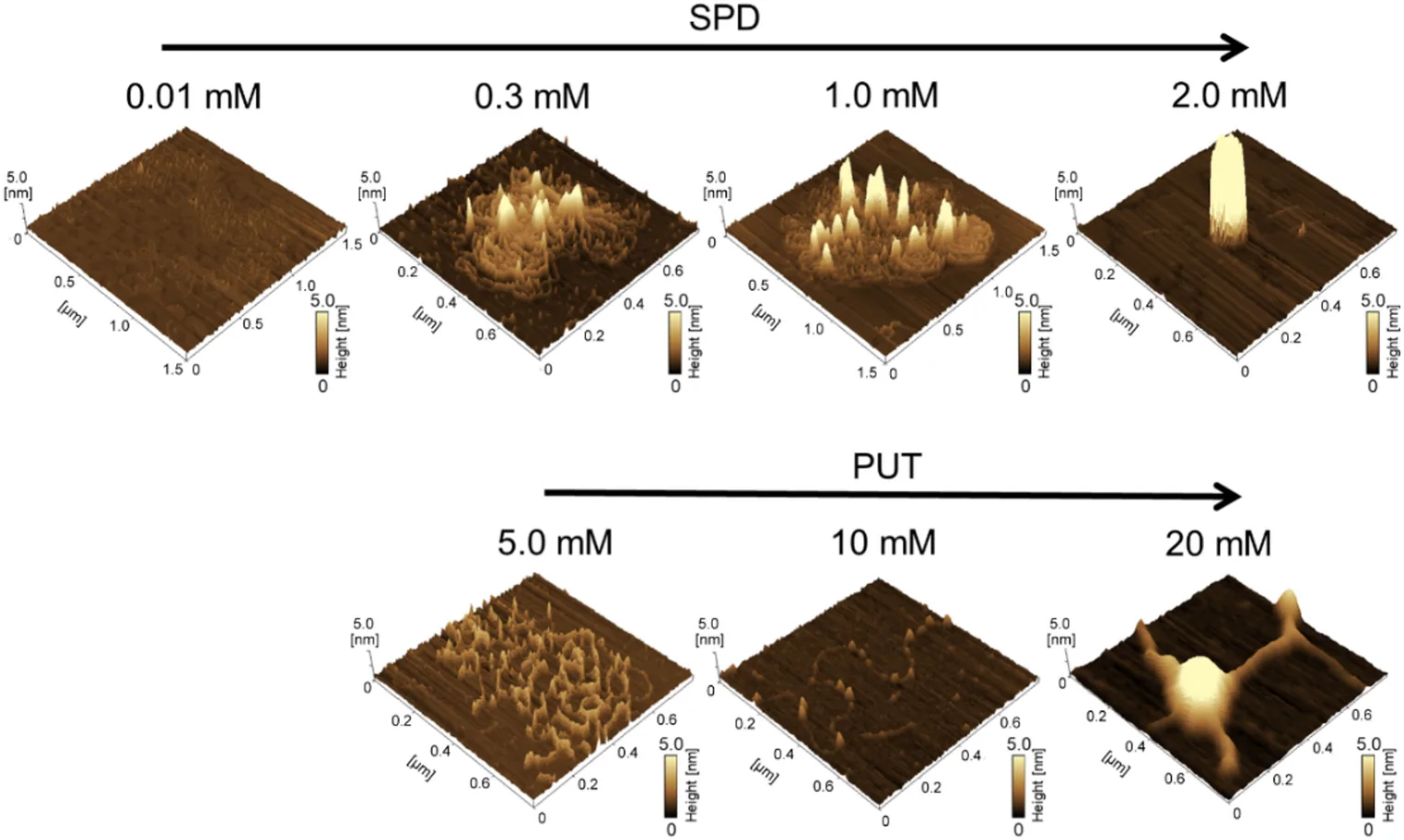
3D AFM images of T4 GT7 DNA at various concentrations of SPD and PUT

In addition, with increasing SPD concentration, the number of dense DNA core regions increased, the height of the core gradually increased, and the DNA eventually folded into a tightly compacted single core at 2.0 mM SPD (Fig. 6). In this state, the access of RNA polymerase to DNA is likely restricted because of the highly dense packaging, despite the almost perfect neutralization of the negative charge on the DNA strands, resulting in the inhibition of gene expression. This interpretation is consistent with the results shown in Fig. 1(a), where gene expression was completely inhibited at 2.0 mM SPD.

In contrast to SPD, PUT did not induce tight compaction of DNA, even at high concentrations. This observation is consistent with previous reports showing that diamines with an even number of carbon atoms, including PUT, have little effect on DNA compaction (Yoshikawa and Yoshikawa 1995). On the basis of our quantitative analysis (Fig. 5(b), compared with the coil state obtained at 0.01 mM SPD, the mesh-like and partially thick structures induced by 5.0 or 10 mM PUT exhibited approximately 0.6- to 0.7-fold lower segmental density (*L*/*S*) values indicating that the DNA chains remained in an unfolded conformation. In contrast, the crossing probability, *ξ*, of the DNA chains did not notably increase with 5.0 or 10 mM PUT and clearly differed for the DNA samples in the presence of 0.3 or 1.0 mM SPD. The spatially expanded conformation of DNA in the presence of PUT likely increases the effective volume over which the RNA polymerase encounters and binds to the DNA strand. In addition, under relatively low crossing probability, RNA polymerase may be allowed to move along the DNA strand smoothly. Under such conformational characteristics of DNA, transcriptional activity is promoted by the addition of relatively low concentrations of PUT (Fig. 1(b). Regarding the inhibitory effect of PUT at higher concentrations, the formation of kinked structures at high concentrations of PUT is expected to play an important role. Recently, Ogawa et al. (2024) reported that the anticancer drug hydroxyurea caused partially thick and kinked-branching structures through the promotion of torsional structure formation in DNA while maintaining the B-type structure. Such a conformational change increased the rigidity of the DNA conformation and led to the inhibition of gene expression (Ogawa et al. 2024).

A distinct difference between SPD and PUT is that the latter induces torsion in DNA strands. These localized twists in the DNA strands caused by PUT likely contribute to the formation of a mesh-like structure that progressively transitions into a partially thick conformation (Fig. 3(e)–(g). Owing to its right-handed helical geometry, double-stranded DNA tends to writhe in the left-handed direction upon bending (Yanao et al. 2015). Additionally, polyamines can cause local distortions in the DNA helix, suggesting that such interactions may induce unique mesh-like structures in the presence of PUT (Pastré et al. 2006). Many studies have revealed that polyamines, such as SPD and PUT, bind to the major groove, minor groove, and phosphate groups on the basis of simulations and spectral analysis of short DNA strands, such as calf thymus DNA (Brysont and Greenall 2000; Ouameur and Tajmir-Riahi 2004; Bignon et al. 2017). However, the relationships between these binding modes and their effects on higher-order structures of genome-sized DNA remain largely unexplored. Through single-molecule observations of genome-sized DNA using AFM, we previously demonstrated that polyamines can induce various higher-order DNA conformations and that linear polyamines tend to induce flower-like structures (Muramatsu et al. 2016; Nishio et al. 2018, 2019, 2023; Tanaka et al. 2020; Kitagawa et al. 2021). On the basis of these considerations, despite its linear structure, PUT is assumed to induce the formation of a mesh-like structure accompanied by localized DNA torsion.

### Quantitative evaluation of the higher-order structural change in DNA caused by the coexistence of SPD and PUT

**Fig. 7:**
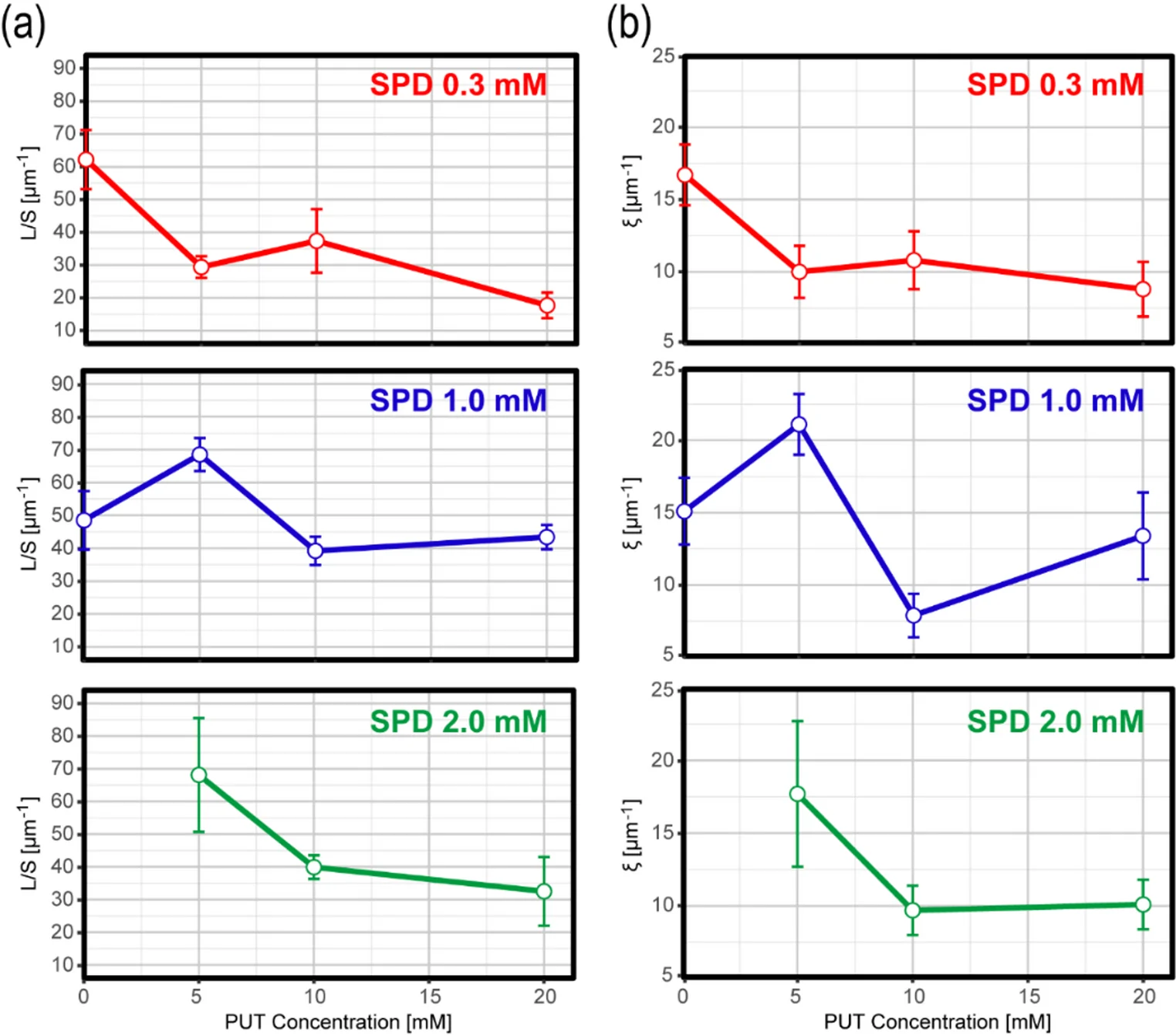
Changes in the (**a**) *L*/*S* (the segmental density) and (**b**) *ξ* (crossing probability) with the PUT concentration at various concentrations of SPD. The average values with SD obtained from the analyses of three individual DNA molecules, which have a major structural feature under each condition, were plotted

To quantitatively evaluate the manner of change in the higher-order structure of DNA, depending on the concentrations of coexisting SPD and PUT (as shown in Fig. 4), we measured the segmental density, *L*/*S*, and crossing probability, *ξ*, on the basis of the obtained AFM images. The changes in the *L/S* and *ξ* values are shown in Fig. 7, including the data in the presence of SPD or PUT alone, as shown in Fig. 5(b). In the bottom panel of Fig. 7(a, b), we show no data points when [PUT] = 0 mM because of the formation of an aggregated/condensed state at [SPD] = 2.0 mM. The top graphs in Fig. 7(a, b) reveal that when [SPD] = 0.3 mM, both characteristic parameters, the *L/S* and *ξ*, decrease with the coexistence of PUT. A similar decreasing trend in the effect of PUT is observed for the data at [SPD] = 2.0 mM (refer to both graphs in Fig. 7(a, b)). In contrast, a bimodal effect of PUT on these parameters is observed when [SPD] = 1.0 mM (middle graphs in Fig. 7(a, b)). Interestingly, the gene expression activity also exhibited a similar bimodal promotion and inhibition effect with increasing PUT concentration in the presence of 0.3 and 0.5 mM SPD, as shown in Fig. 2. Here, it is noted that the bimodal effect observed in the present study is a reliable and substantially sufficient difference beyond the margin of error.

Although the SPD concentrations revealing the bimodal effect differ between the gene expression activity and higher-order structure parameterized by *L/S* and ξ, such a small difference for the coexisting concentrations of PUT and SPD to generate the bimodal effect of PUT can be attributed to the difference in the buffer conditions between cell-free gene expression and AFM measurement.

**Fig. 8:**
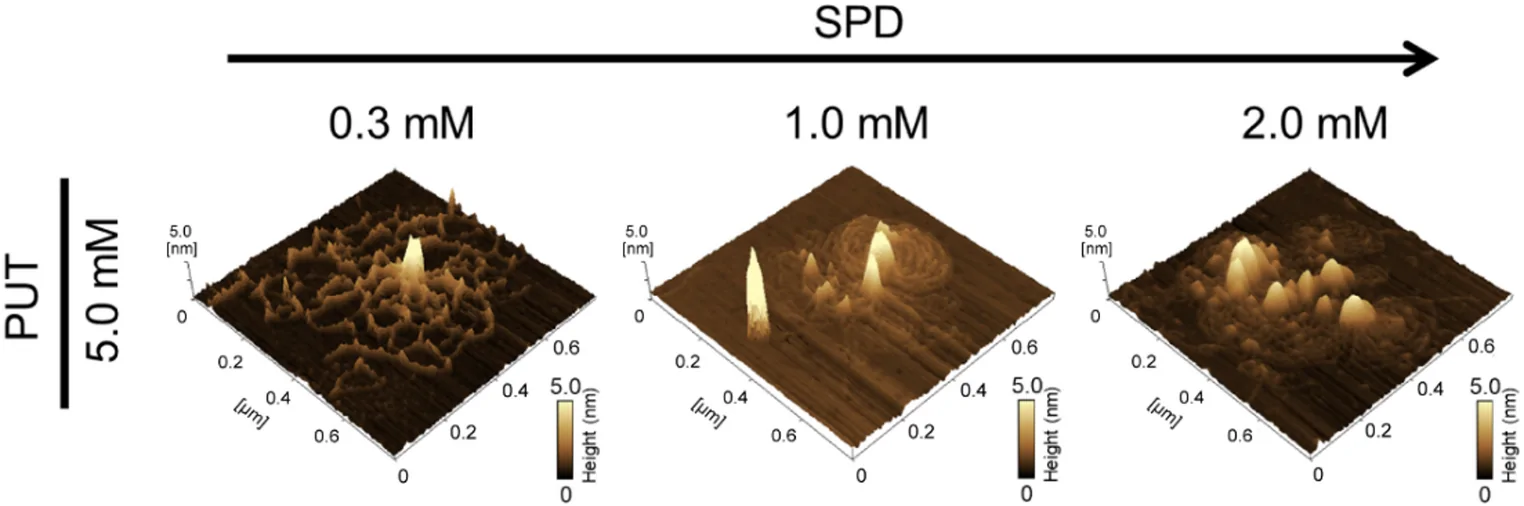
3D AFM images of T4 GT7 DNA in the presence of various concentrations of SPD at a fixed concentration of 5 mM PUT

As mentioned above, we found that PUT caused the unfolding of the compact DNA caused by SPD, yielding the formation of a mesh-like structure. As shown in Fig. 1(b), compared with the flower-like structure, this mesh-like structure resulted in higher gene expression activity. Not only the effects on the higher-order structural changes of DNA but also the difference in concentration range are likely to affect gene expression, as PUT does not exhibit a pronounced expression-enhancing or expression-suppressing effect until its concentration is 10 times greater than that of SPD application. Furthermore, the addition of PUT in the presence of SPD at concentrations that promoted gene expression resulted in either gene expression enhancement or suppression, depending on the PUT concentration (Fig. 2). As shown in Figs. 4 and 8, the DNA compacted at higher SPD concentrations was less efficiently unfolded by PUT, indicating that increased SPD levels restrict PUT-induced unfolding. As a result, the increase in gene expression at 0.5 mM SPD did not reach the level observed at 0.3 mM SPD or with PUT alone, which is likely due to limited structural transition caused by stronger compaction (Fig. 2). At even higher concentrations, although a fully condensed DNA structure was observed in the presence of 2.0 mM SPD, the genome-sized DNA compaction structure was unfolded due to the PUT reaction, showing a trend similar to that of 1.0 mM SPD (Yoshikawa and Yoshikawa 1995; Ogawa et al. 2024).

## Discussion

With respect to the effect of polyamines on DNA, the attractive interaction of polyamines with DNA double-strands intensifies with increasing positive cationic number, whereas the stability of the polyamine–DNA complex increases with increasing charge (Ouameur and Tajmir-Riahi 2004; Francesca Mocci 2021). Additionally, an increase in the polyamine concentration causes a conformational transition of DNA from an elongated coil state to a compact state, and a further increase in its concentration induces a reentrant transition back to an elongated state again (Pelta et al. 1996; Murayama et al. 2003; Carlstedt et al. 2012). In addition to the effects of polyamines on DNA, in this study, we observed both synergistic and antagonistic effects between polyamines with different cationic numbers. We previously proposed a model based on the counterion condensation theory that incorporates the contribution of the translational entropy of counterions to explain the antagonistic effects of Mg(2+) and SPD(3+) on higher-order structural transitions of DNA (Tongu et al. 2016). For the compaction of DNA with SPD(3+) in the absence of Mg(2+), an increase of translational entropy due to the release of monovalent condensed counterions into the bulk solution through an ion exchange by the added SPD(3+) contributes to stabilization of the compact state of DNA (Takahashi et al. 1997; Yoshikawa and Yoshikawa 2002). In contrast, the presence of Mg(2+) decreases the gain of entropy contribution by the ion-exchange between monovalent and SPD(3+). In this model, we explained that the presence of Mg(2+) decreases the translational entropy gain associated with ion exchange between monovalent and trivalent cations, leading to the unfolding of DNA condensed by SPD. The addition of monovalent cations, such as Na(1+) and K(1+), in the presence of SPD induces the unfolding transition of DNA through counterion exchange (Zinchenko and Yoshikawa 2005; Hibino et al. 2006; Nishio et al. 2020). Therefore, the observed unfolding of the flower-like structure can be attributed to the partial replacement of SPD with PUT, which does not induce notable DNA compaction. Interestingly, with respect to the effect on gene expression, the coexistence of SPD and Na(1+) or K(1+) has been reported to enhance gene expression further beyond the maximum level observed with SPD alone (Nishio et al. 2020). According to standard knowledge of the physical chemistry of aqueous solutions, as in the Debye–Hückel theory (Sastre de Vicente 2004; Robson Wright 2007), in an aqueous solution, each ion is surrounded by a cloud of counterions that shield its charge. On the basis of this theoretical framework, the shielding effect was estimated in terms of the ionic strength *I* as follows:

$$\:I=\frac{1}{2}{\sum_{i=1}^n}{c}_{i}{z}_{i}^{2}$$

where $\:{c}_{i}$ is the molar concentration of ion *i*, $\:{z}_{i}$ is the charge number of the ion, and the sum is taken from all the different ions (*n*) in the solution. Therefore, the shielding effect of the counterions is additive among the influences of different ions when the square of the ionic valence is included. Such an evaluation of the shielding effect is based on the Debye–Hückel-type mean-field assumption, which neglects multi-ion correlations. For double-stranded DNA, the distance between neighboring negatively charged phosphates is approximately 4.2 times greater than the Bjerrum length (Manning 1969; Russell 1971; O’Shaughnessy and Yang 2005); $\:{\mathcal{l}}_{B}={e}^{2}/\epsilon\:kT$, where *e* is the electron charge, $\:\epsilon\:$ is the dielectric constant, and *kT* is the thermal energy. The Bjerrum length is the distance at which the electrostatic energy between two monovalent charges is equivalent to the scale of thermal energy, and $\:{\mathcal{l}}_{B}$ ≈ 0.7 nm is obtained in an aqueous environment. Therefore, approximately 75% of the negative phosphate charges along the double-stranded DNA are neutralized by counterions in an aqueous solution containing monovalent cationic species. Importantly, counterion condensation theory (Manning 1969; Russell 1971; O’Shaughnessy and Yang 2005) implies a competitive effect between cationic ions with different valences. In relation to such a competitive effect on the binding between cations of different valences, we reported (Tongu et al. 2016) that divalent cations, i.e., Mg(2+) and Ca(2+), inhibit DNA compaction induced by a trivalent cation, SPD. In the absence of SPD, divalent cations cause DNA shrinkage. Such competitive effects between 2 + and 3 + cations have been theoretically interpreted in terms of changes in the translational entropy of the counterions (Tongu et al. 2016). The competitive effect between SPD and PUT observed in this study can be explained by essentially the same theoretical assumption regarding the change in translational entropy of counterions with different valences.

Next, we examine the effects of coexisting polyamines on gene expression. In the measurements in which PUT and SPD do not coexist, in terms of gene expression activity, the effects of both PUT and SPD on gene expression, i.e., promotion and inhibition, are bimodal and depend on their concentrations. The maximum degree of promotion by PUT is almost an order of magnitude greater than that of SPD, and the concentration required to maximize the activity by PUT was approximately one order of magnitude greater than that of SPD. Interestingly, in the presence of PUT and SPD, the maximum promotion of gene expression by PUT was further enhanced by a small amount (0.3 mM) of SPD (Fig. 2). In contrast, in the presence of a larger amount of SPD, the maximum promotion activity tended to decrease, as did the inhibitory effect of SPD against PUT.

With respect to the change in the higher-order structure of DNA for the experiments with PUT or SPD alone, SPD generates a flower-like conformation (Muramatsu et al. 2016) at lower concentrations and causes a tightly compact state at higher concentrations, whereas PUT induces the formation of a mesh-like structure at lower concentrations (Fig. 3(e) and causes a kinked conformation with small cores at higher concentrations. The generation of a mesh-like conformation in the presence of various concentrations of PUT was promoted in the presence of a small amount (0.3 mM) of SPD (Fig. 4(a)–(c). Notably, with the coexistence of PUT and SPD at suitable concentrations, the conditions needed for the polyamines to generate a mesh-like structure correspond to the solution conditions that increase gene expression. Additionally, the solution conditions that cause tightly packed and condensed states (Fig. 6) correspond to those that inhibit gene expression. As shown in Fig. 7, the observed synergistic (cooperative) and antagonistic (anti-cooperative) effects between PUT and SPD were closely related to the changes in the degree of DNA compaction or condensation depending on the mutual concentrations of PUT and SPD under coexisting conditions.

The synergistic and antagonistic effects of PUT and SPD on higher-order structural changes in DNA can be interpreted in terms of the complex correlations among charged species in aqueous environments. As DNA is a highly charged polyelectrolyte, counterions localize near the array of negatively charged phosphate groups along the double-stranded structure, and multivalent cations, such as PUT and SPD, interact with the phosphate groups to cause the release of counterions that condense around the double helix; that is, counterion condensation theory (Manning 1969; Russell 1971; O’Shaughnessy and Yang 2005). Therefore, PUT and SPD bind to the aligned phosphate groups in DNA as mutual competitors. Here, changes in the higher-order structure of genome-sized DNA are closely related to changes in the effective negative charge through the binding of counterions by modifying the manner of interaction between segments along long DNA chains  (Yoshikawa, et al. 1995; Takahashi et al. 1997; Yamasaki et al. 2001; Zinchenko, et al. 2005; Hibino et al. 2006; Muramatsu et al. 2016; Tongu et al. 2016; Nishio et al. 2019, 2023; Ogawa et al. 2024). In contrast, under solution conditions with negligible changes in the higher-order structure of DNA, both PUT and SPD exhibit an increase in electronic shielding because they act as mutual cooperators as cationic species, following the general framework of Debye–Hückel.

It is expected that the decrease of negative charge for the shrunken state of DNA in the presence of suitable amounts of polyamines is associated with the promotion of gene expression. It is known that RNA polymerase is negatively charged in a usual aqueous environment of living systems (Chelm and Geiduschek 1979; De Carlo et al. 2003; Garrett R. H. 2023). Thus, it is expected that polyamines will generate favorable conditions for RNA polymerase to access DNA segments with a reduced negative charge. In addition, substrates for the transcriptional reaction, i.e., NTPs, are also negatively charged. Thus, we may expect the promotion of transcriptional reaction for the shrunken DNA. On the other hand, a tightly compacted state of DNA rejects the access of RNA polymerase and NTPs, exhibiting complete inhibition of transcription. Concerning the shrunken conformation of DNA, it was found that the mesh-like conformation is more favorable for gene expression compared to the flower-like conformation. Such a favorable effect on the mesh-like conformation would be regarded as related to a small modification of the secondary structure of the double-stranded DNA as a possible mechanism (Kornyshev 2010; Yanao and Yoshikawa 2014).

This study demonstrates that switching between synergistic and antagonistic interactions depends on environmental conditions, even for very simple cationic chemical species with different valences. Similar bimodal interactions should occur with polyamines in the complex inner environment of living cells. Several publications have described the bimodal effects of activator/inhibitor of several biological species in living systems (Calabrese 2013; Ramesh and Krishnan 2023). Further studies accounting for the possible bimodal/opposite effects on biomolecular interactions could provide new perspectives/hypotheses (Yoshikawa 2002) for the working mechanisms of complex networks with thousands of biological species in living matter.

## Materials and methods

### Chemicals

Spermidine trihydrochloride (SPD) was purchased from Nacalai Tesque (Kyoto, Japan). Putrescine dihydrochloride (PUT) was purchased from Wako Pure Chemical Industries (Osaka, Japan). Tris-hydrochloride acid buffer (Tris-HCl; pH 7.4) and T4 GT7 DNA (166 kbp, contour length 57 μm) were purchased from Nippon Gene (Tokyo, Japan). Plasmid DNA (luciferase T7 control DNA, 4331 bp) containing the firefly luciferase gene was purchased from Promega (Madison, WI, USA).

### Luciferase assay for gene expression

A cell-free luciferase assay was conducted using the TnT (rabbit reticulocyte lysate) T7 Quick Coupled Transcription/Translation System (Promega) in accordance with the manufacturer’s instructions and previous reports (Kanemura et al. 2018; Nishio et al. 2019, 2020, 2023; Kitagawa et al. 2021; Ogawa et al. 2024). Plasmid DNA (4331 bp) encoding a firefly luciferase gene with a T7 promoter sequence was used as the DNA template at a final concentration of 0.6 µM in nucleotide units in the reaction mixture. A 25-µL reaction mixture was prepared containing 18.5 µL of TNT^®^ Quick Master Mix, 20 µM methionine, and desired concentrations of polyamines. The reaction mixture containing the DNA was incubated for 90 min at 30 °C with various concentrations of SPD and PUT using a Dry Thermo Unit (TAITEC, Saitama, Japan). We measured the effects of SPD and PUT addition on the luminescence intensity (luciferase expression). Their intensities were evaluated following the addition of luciferase assay substrate and buffer (luciferase assay reagent, Promega) by detecting the emission intensity at approximately 565 nm using a luminometer (MICROTEC Co., Chiba, Japan). The intensity was normalized to the control condition (in the absence of SPD and PUT) as 1.00. For the measurements shown in Fig. 2, SPD was added to the reaction mixture containing template DNA, followed by PUT.

### AFM observation

AFM images were obtained using a SPM-9700 scanning probe microscope (Shimadzu, Kyoto, Japan). T4 GT7 DNA (0.6 µM) was dissolved in buffer (10 mM Tris-HCl at pH 7.4, with various concentrations of SPD (0.01–2.0 mM) and PUT (5.0–20 mM)), incubated for 10 min at room temperature (24 °C), and then transferred onto a freshly cleaved mica surface. The samples were then rinsed with ultrapure water (Milli-Q) and dried with pure nitrogen gas to prevent humidity-induced changes in DNA morphology and water contamination. After drying, samples were immediately placed on an AFM scanner and observed with tapping mode in air. We imaged the mica surface and conducted all the measurements in the tapping mode with AFM in air. The cantilever (OMCL AC200TS-C3; Olympus, Tokyo, Japan) was 200 μm long with spring constants ranging from 9 to 20 N/m. The scanning rate was 0.4 Hz, and images were obtained using the height mode in a 512 × 512-pixel format. The obtained images were plane-fitted and flattened using the computer program supplied by the imaging module. Three-dimensional (3D) images of DNA were obtained from the DNA height information (256 × 256-pixel format) using a computer program supplied with the imaging module.

### Quantitative evaluation of the higher-order structural change of DNA from AFM images

We evaluated the DNA segmental density (*L*/*S*) and crossing probability (*ξ* = *N*/*L*, µm^− 1^) from the AFM images. As shown in Fig. 5(a), we manually traced the DNA (green lines) in the fixed area (*S*, µm^2^, green dotted area), which is a partial region of the entire DNA molecule other than the highly condensed core regions. Afterward, we measured the total DNA length (*L*, µm) and counted the total number of crossings (*N*, blue dots). We analyzed three individual DNA molecules (see Fig. S1 and S2) that had the major structural features for each condition. The average values with SDs are summarized in Fig. 5(b) and Table S1 in the supplementary information. ImageJ (ver. 1.54p) was used for AFM image analysis.

## Supplementary Information

Below is the link to the electronic supplementary material.


Supplementary Material 1

