# Supplementary material for: Effects of synergistic/antagonistic interactions between spermidine (3+) and putrescine (2+) on gene expression through higher-order DNA structural modification
Source: Eur Biophys J. 2026 Mar 2;55(4):607–19. doi: 10.1007/s00249-026-01831-w (PMC13521979; doi:10.1007/s00249-026-01831-w)
Supplement: Supplementary file 1 [file 249_2026_1831_MOESM1_ESM.pdf]

Supplementary information

Effects of Synergistic/Antagonistic Interactions between Spermidine(3+) and Putrescine(2+) on Gene Expression through Higher-Order DNA Structural Modification

Haruto Ogawa[1], Takashi Nishio[2*], Yuko Yoshikawa[1], Takahiro Kenmotsu[1], Kenichi Yoshikawa[1]

1) Faculty of Life and Medical Sciences, Doshisha University, Kyoto 610-0394, Japan.

2) Molecular Biosystems Research Institute, National Institute of Advanced Industrial Science and Technology (AIST), Ibaraki 305-8566, Japan

*Corresponding author's email: nishio-t@aist.go.jp; ORCID: 0000-0001-8761-1776

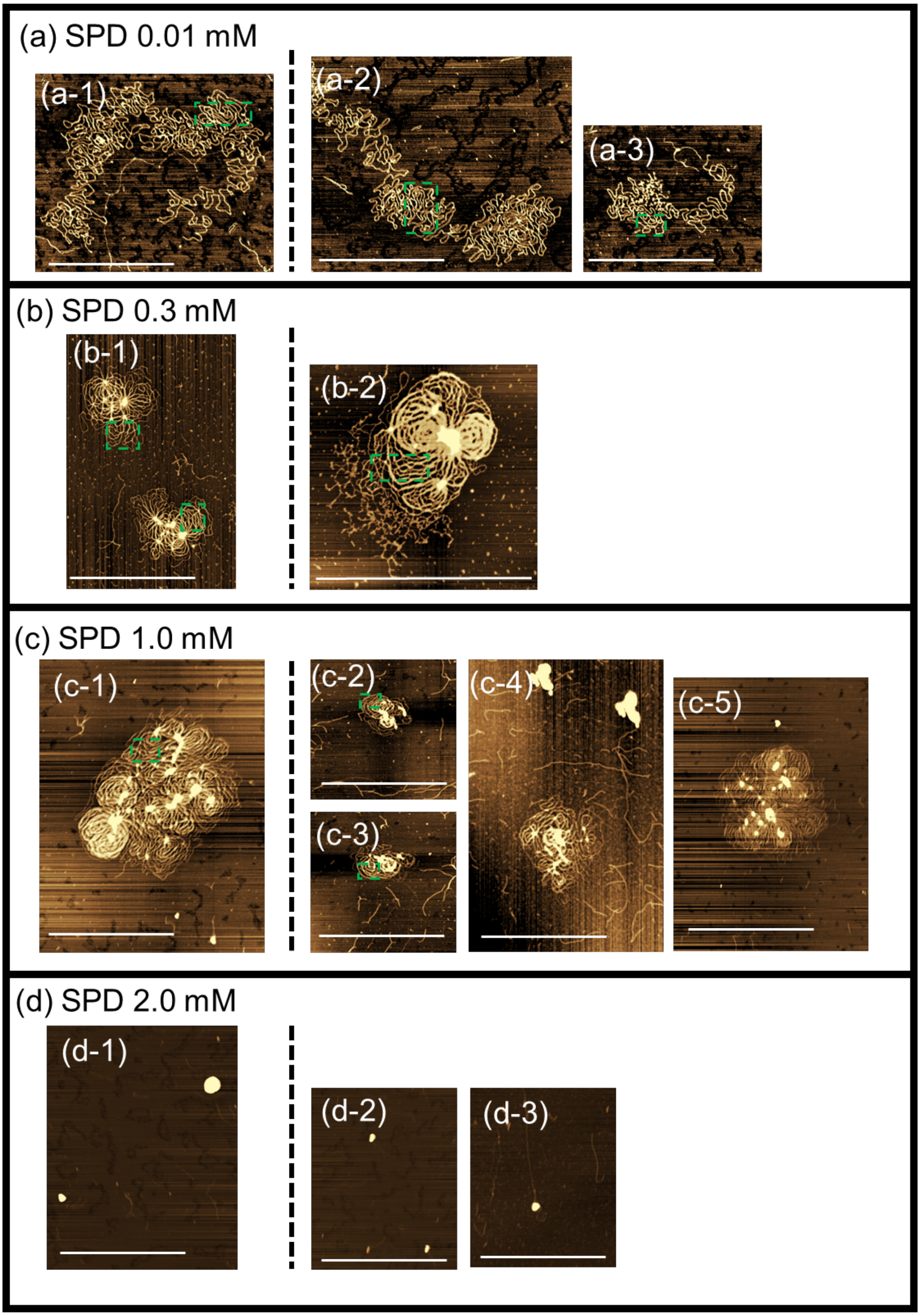

Fig. S1 Additional AFM images of T4 GT7 DNA at various concentrations of SPD and

PUT. The images shown in the left-most column correspond to those in Fig. 3 of the main text. The green dotted squares indicate the regions selected for their similar DNA higher-order structural tendencies and adapted for the evaluation of the parameters L, S, and ξ. In each figure, the scale bar is 1 μm.

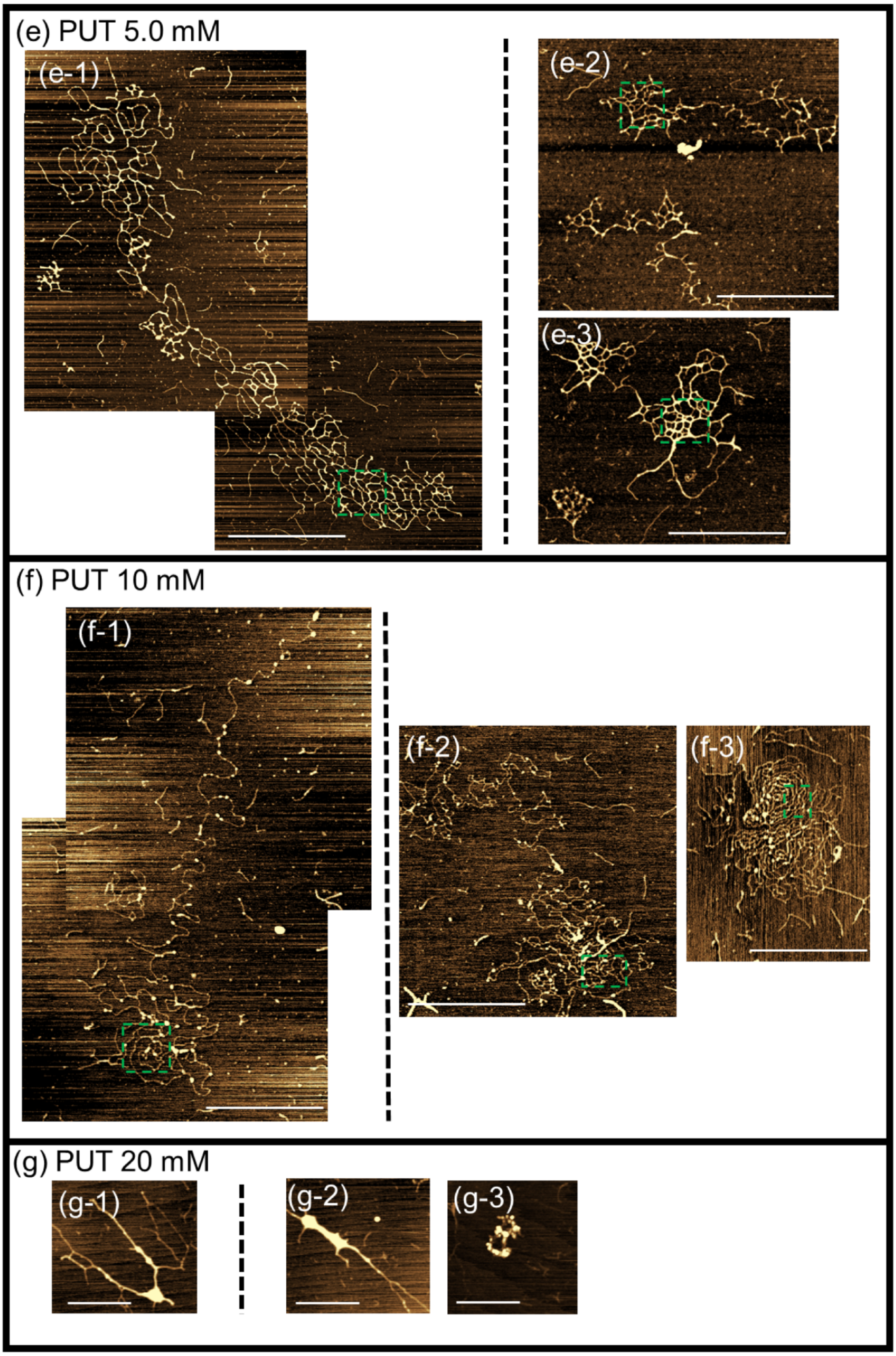

24

25 Fig. S1 (continued)

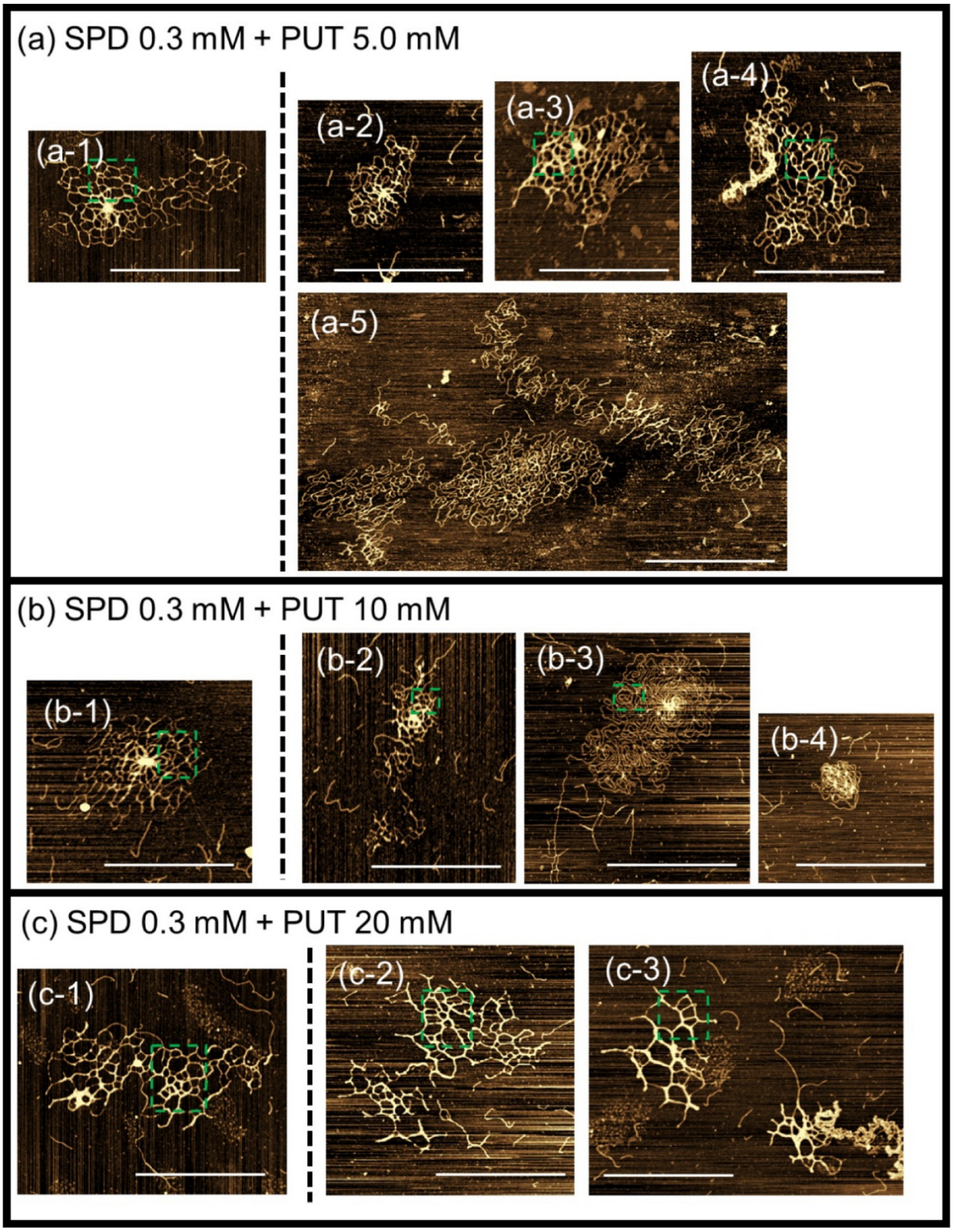

Fig. S2 Additional AFM images of T4 GT7 DNA exposed to both SPD and PUT. The images shown in the left-most column correspond to those in Fig. 4 of the main text. The green dotted squares indicate the regions selected for their similar DNA higher-order structural tendencies and adapted for the evaluation of the parameters L, S, and ξ. In each

figure, the scale bar is 1 μm.

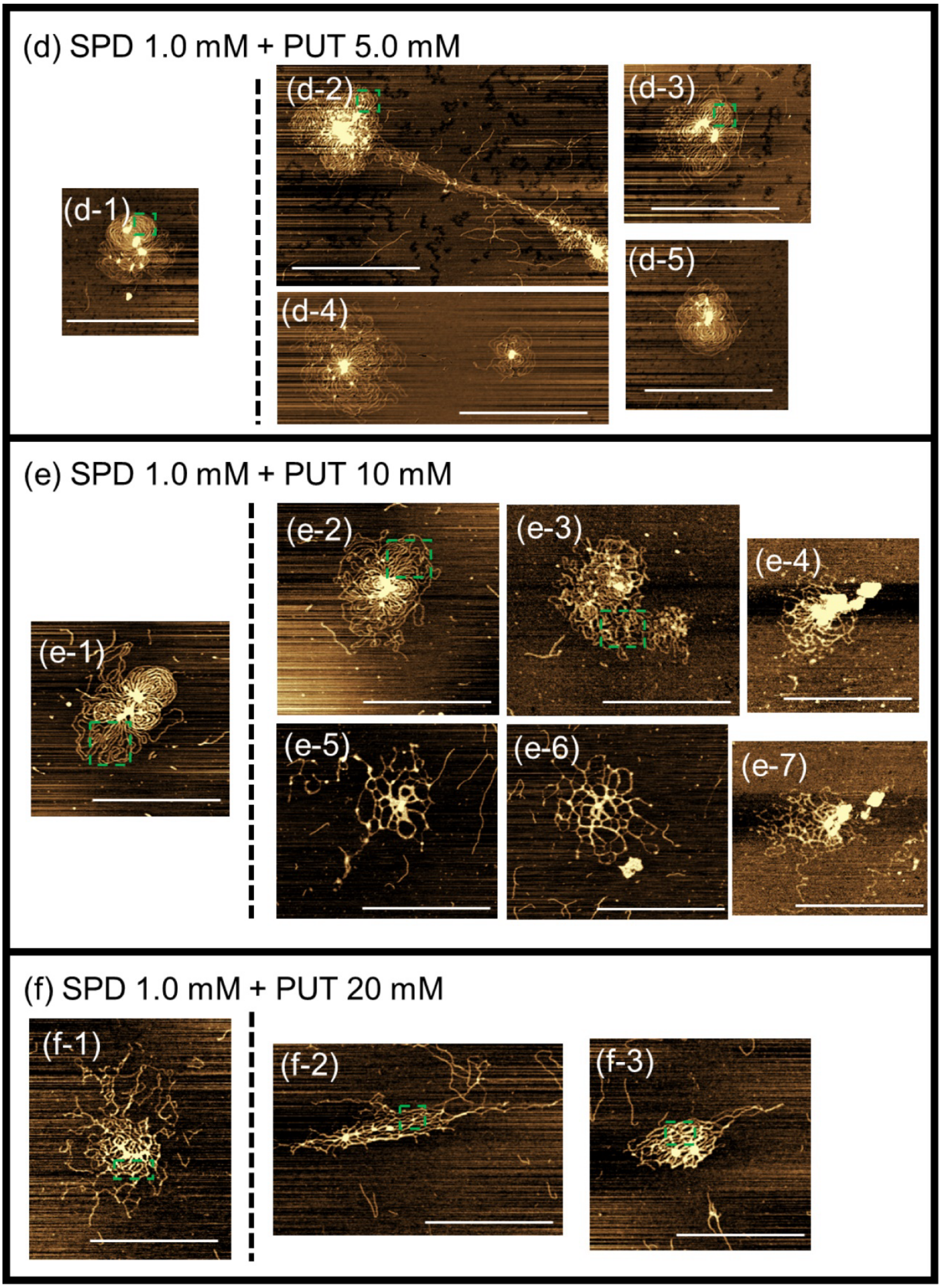

Fig. S2 (continued)

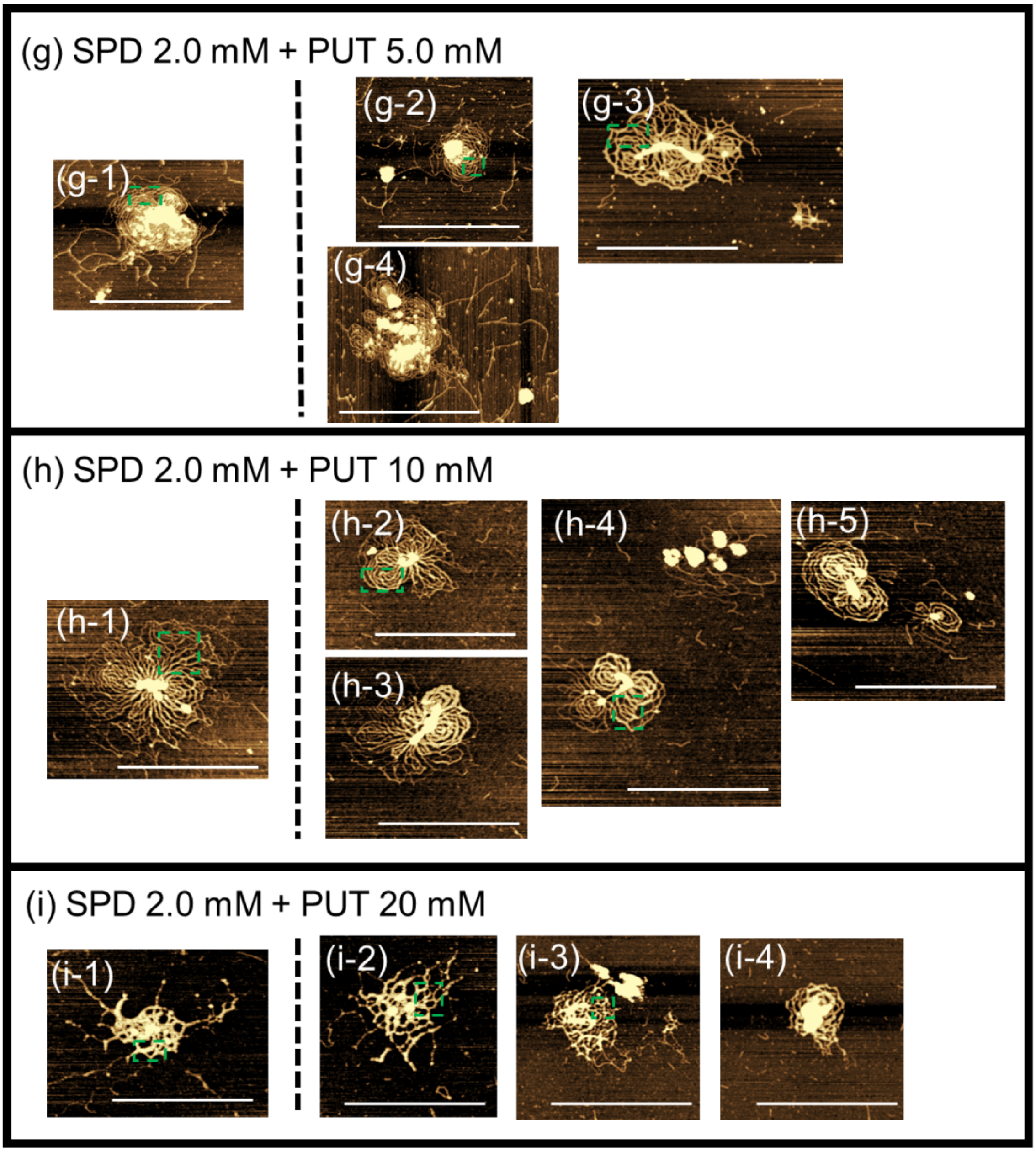

Fig. S2 (continued)

Table S1. Effective segmental density, L/S, and probability of crossing, ξ, under the

coexistence of SPD and PUT at various concentrations evaluated from the AFM images.

	L/S [μm^{-1}]	ξ [μm^{-1}]
SPD 0.3 mM+ PUT 5.0 mM	29.4 ± 3.3	10.0 ± 1.8
SPD 0.3 mM+ PUT 10 mM	37.4 ± 9.7	10.8 ± 2.0
SPD 0.3 mM+ PUT 20 mM	17.7 ± 3.9	8.8 ± 1.9
SPD 1.0 mM+ PUT 5.0 mM	68.5 ± 5.0	21.1 ± 2.1
SPD 1.0 mM+ PUT 10 mM	39.2 ± 4.3	7.9 ± 1.5
SPD 1.0 mM+ PUT 20 mM	43.4 ± 3.7	13.4 ± 3.0
SPD 2.0 mM+ PUT 5.0 mM	68.1 ± 17.4	17.7 ± 5.0
SPD 2.0 mM+ PUT 10 mM	39.9 ± 3.6	9.7 ± 1.7
SPD 2.0 mM+ PUT 20 mM	32.5 ± 10.5	10.1 ± 1.7

L : total length [μm], S : area [μm^2], N : number of crossings,
$\xi = N / L$ [μm^{-1}] : crossings probability
